# Real-Time Risk Assessment Detection for Weak People by Parallel Training Logical Execution of a Supervised Learning System Based on an IoT Wearable MEMS Accelerometer

**DOI:** 10.3390/s23031516

**Published:** 2023-01-30

**Authors:** Minh Long Hoang, Armel Asongu Nkembi, Phuong Ly Pham

**Affiliations:** 1 Department of Engineering and Architecture, University of Parma, 43124 Parma, PR, Italy; 2 Department of Industrial Engineering, University of Salerno, 84084 Fisciano, SA, Italy

**Keywords:** machine learning, IoT, wearable device, random forest classification, accelerometer, activity recognition, COVID-19

## Abstract

Activity monitoring has become a necessary demand for weak people to guarantee their safety. The paper proposed a Parallel Training Logical Execution (PTLE) system using machine learning (ML) models on a microelectromechanical system (MEMS) accelerometer to detect coughs, falls, and other normal activities. When there are many categories, the ML prediction can be confused between these activities with each other. The PTLE system trains several models in parallel with more specific activity classes in each dataset. The shared tasks between parallel models relieve the complexity for a single one. There are six additional parameters for accelerometer characteristics, which were calculated from three axes accelerations as input features to improve the ML’s consciousness. Once all models were trained, the system was ready to receive the input accelerations and activated the logical flow to manage link operation between these ML models for output predictions. Random Forest (RF) had the highest potential among the ML classification algorithms after the validation. In the experiment, the comparison between the PTLE model and the regular ML model were carried out with real-time data from an M5stickC wearable device on the user’s chest to the trained models on PC. The result showed the advancement of the proposed method in term of precision, recall, F1-score with an overall accuracy of 98% in the real-time test. The accelerations from the wearable device were sent to ML models via Wi-Fi with Message Queue Telemetry Transport (MQTT) broker, and the activity predictions were transferred to the cloud for the family members or doctor care based on Internet of Things (IoT) communication.

## 1. Introduction

Nowadays, many frailty/old people need to be under health monitoring at home. Activity recognition is an important aspect of context-aware systems. Cough and falls are two of the most anxious events which need to be detected for health’s sake. Cough is a symptom of many types of flu or coronavirus disease 19 (COVID-19) [1]. Suppose a coughing fit is severe or lasts for an extended period. In that case, components of the respiratory system and other body areas can be damaged, such as blood vessels, chest or muscle pain, etc. [2]. Falls typically occur unexpectedly while performing daily activities and are a health threat, especially for adults of age 65 and older [3]. Other daily activities such as sitting, walking, and sleeping can also be monitored to understand the typical behaviors of weak people.

The MEMS accelerometer [4,5,6] has a high potential capacity to support activity classifications. The accelerations on the X-axis, Y-axis, and Z-axis vary depending on body movement, which can be a key factor in providing valuable information for action recognition. There are articles that research accelerometers in human activity recognition (HAR) by deep learning (DL), a subset of ML. In the paper [7], the research focused on a hybrid deep learning model that takes heterogeneous sensor data, an acceleration sensor, and an image as inputs. Another work [8] classified two different physical activities, viz., walking and brisk walking, with deep neural networks from mobile phone sensors such as accelerometers, gyroscopes, magnetometers, etc. Although these approaches can achieve impressive accuracy, deep learning requires a large amount of data from multiple sources to classify activities. The requirement of expensive GPUs and complex designs can increase the cost and difficulty to the users.

On the other hand, supervised ML has a good capability to recognize human activity from accelerometers with less complexity. Various papers evaluate supervised classification algorithms with inertial sensors [9,10,11]. Generally, the ML algorithms are compared, and then the most suitable model is selected for the main prediction [12]. However, it is also necessary to enhance the accuracy of the fittest model. Otherwise, support from more accelerometers on different body parts will be needed [13]. Most presented works used more than two sensors which do not boost the HAR results considerably, as described by Bao and Intille [14] and Olguín and Pentland [15]. In real life, it is bothersome for the users to wear multiple sensors on their bodies daily, which can make them uncomfortable, and the cost will also increase. 

Therefore, this paper proposed the PTLE technique, which trains several models in parallel with reduced activity categories in each dataset. With less complex computation, the prediction apparently reaches better precision from single tri-axial accelerometers. The dataset can be fed into the parallel models in multiple classification cases to optimize the ML model’s accuracy. Practically, with a greater number of classes or labels present in the training dataset, it is more challenging for the ML model to predict the proper outputs. In this case, there are five activities: cough, fall, sit, walk, and sleep. Among these actions, cough and fall are the abnormal type; sit, walk and sleep belong to the normal type. Hence, the first step is to use ML model to classify them into two separated models. One model was trained with the ‘cough–fall’ dataset, while the other was trained with the ‘sit–walk–sleep’ dataset. These data were extracted from the original dataset but now contain different labels.

In addition to the accelerations of the three axes, this research utilized six other significant features of accelerometer characteristics that were calculated to improve the distinguished ability of the ML models as follows:

- Xacc, Yacc, and Zacc are the acceleration of the X-axis, Y-axis, and Z-axis, respectively.
- ΔXacc, ΔYacc, and ΔZacc are the absolute difference values between two consecutive samples of the X-axis, Y-axis, and Z-axis, respectively.
- Δacc_Norm is the magnitude normalization of Δ acceleration.
- ΔRoll and ΔPitch Zacc are the absolute difference values between two consecutive samples of roll and pitch, respectively.

After validation, the RF algorithm accomplished the best accuracy score among the used algorithms, so it was implemented into the ML models. 

With the support of IoT [16], the accelerations from the wearable device on the chest were transferred to the ML models via MQTT broker based on Wi-Fi. Then, ML predictions were sent to the IoT dashboards for healthcare monitoring by family members or doctors. 

The paper is organized as follows: the first part briefly describes the regular ML model. The following parts discuss the PTLE system in terms of training process and logical execution. In the experiment, a real-time comparison between the regular model and the PTLE technique was performed regarding precision, recall, F1-Score, and accuracy. Finally, the conclusion summarizes the work’s achievements and future proposals. 

## 2. Related Work

The PTLE contains the ML models that enable a new approach to utilize various ML algorithms in one method. A published article [17] discussed a parallel architecture to combine traditional and deep learning pattern classification algorithms, for accrued computational and classification accuracy, as an ensemble learning architecture. Generally, the authors used three pipelines to obtain a prediction vector for the same training dataset. The final classification result was based on the majority voting for decision fusion. This work still requires the convolutional neural network (CNN), the multivariate time series classification task, which considerably increases the complexity of the system with high-cost computation/design for the convolution layer and the pooling layer. This approach constitutes a long training time with the large number of weight updates and two other models for the same amount of training data and same classification number. 

MEMS accelerometers [18,19,20,21] have played an essential role in the HAR due to their well-integrated capability inside wearable devices. The acceleration data demonstrate the corresponding fluctuation with the human motion that is extremely useful to combine with the ML approach for monitoring the activity of the weak/old people from a distance. After the training process, ML classifies the action based on input accelerations.

Various types of devices have been used in HAR [22]. In [23], an automatic detection of physical activity (PA) system consisted of five accelerometers and a heart rate monitor based on the Polar chest strap (Wearlink). Another heavy recording device [24] included a chest analog accelerometer device, and wrist accelerations of ADXL202 were used to collect the motion data. Both of these approaches have a decent capacity to achieve real-time data, but multiple sensors can cause additional burdens to users. Other HAR applications employ devices that are embedded in various sensors and have lightweight, portable characteristics. Smartwatches with embedded accelerometers can be used for activity recognition systems that collect data [25,26]. Sensor readings from mobile sensors are also useful for human activity classification [27]. Since these devices are not fixed with to the body’s center, e.g., the chest, it is more challenging to acquire distinctive data between multiple activities. 

There are seven popular algorithms for ML classification: logistic regression (LR), linear discriminant analysis (LDA), K-nearest neighbor classification (KNN), classification and regression trees (CART), Naive Bayes (NB), support vector machines (SVMs), and Random Forest (RF). LG is easy to implement and train efficiently, but it is less effective with non-linear problems because it has a linear decision surface. LDA is also a fast algorithm that uses the mean values of the classes and maximizes the distance between them. However, if the distribution’s mean values are shared between the classes, LDA cannot find a new linearly separable axis. KNN can plug in any distance metric to work with complex objects, but it is a distance-based approach, so the model can be badly affected by outliers, leading to overfitting.

CART is good for interpretation and visual presentation. With automatic feature selection, unimportant features do not impact the result. Nevertheless, it is susceptible and has high variance. A small change in the data can influence the prediction considerably. NB needs less training data to perform better than LR. It assumes that all the features are independent; meanwhile, the features can relate to each other in many practical cases. SVMs work well with a clear margin of separation by using a subset of training points in the decision function (support vectors), which is memory efficient. They have issues with large datasets due to their time-consuming nature, causing reduced prediction quality when there much noise in the data. 

RF is one of the most conceivable algorithms to be implemented into the ML model. Each decision tree generates its prediction by comparing the features of the acquired value with the data of trained nodes. Each node separates the input data into a more specific range. After multiple comparisons, the decision tree provides a prediction. The same progress occurs with other trees. Eventually, the last result comes from the average prediction of all decision trees in the forest. This technique can avoid variation and solve unexpected noise well because each decision tree can act as an analyst [28]. 

The IoT [29] currently has usage cases in primary healthcare for controlling and managing dire situations during unsecured health circumstances such as coronavirus quarantine [30]. MQTT is an outcome and assessment information set (OASIS) standard messaging protocol for the IoT, which has been used widely in the medical monitoring system, as discussed in [31]. In [32], the authors addressed the design MQTT protocol for temperature control in a warehouse using ESP8266 and Wi-Fi. The obtained data were shared to the Baidu intelligent cloud server using MQTT. 

**Figure 1:**
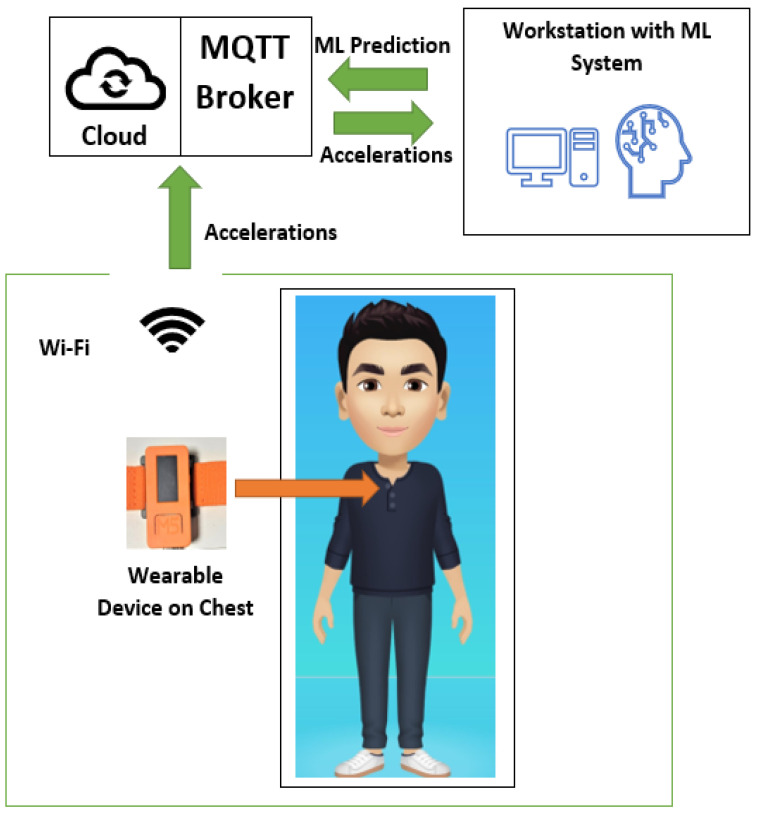
Operation diagram.

In this project, the first model contained all the data, but with only two general classification types. Two following models included the minimized training number with specific classification categories. The final result depends on the logic execution, flowing from the first model to the others instead of majority voting decision. In this way, all models can have supervised learning with ML algorithms without neural network support thanks to the logic flow between models with specific training ranges. In addition, all ML algorithm validation was performed in each model to determine the most suitable one for each ML model. In this application, RF brings the highest score to all, but the results could be different in other applications. Practically, PTLE allows multiple ML algorithms in its system that increase ML flexibility. Furthermore, the logical flow between the trained models leads to high accuracy in the final prediction. This research utilized a M5stickC wearable device, which consists of a microelectromechanical system (MEMS) three-axis accelerometer [33]. The proposed design used the MQTT protocol for ESP32 [34] and a PC for messaging transfer between the wearable device-cloud-ML system, as demonstrated in Figure 1. 

## 3. Materials and Methods

### 3.1. Data Acquisition 

**Figure 2:**
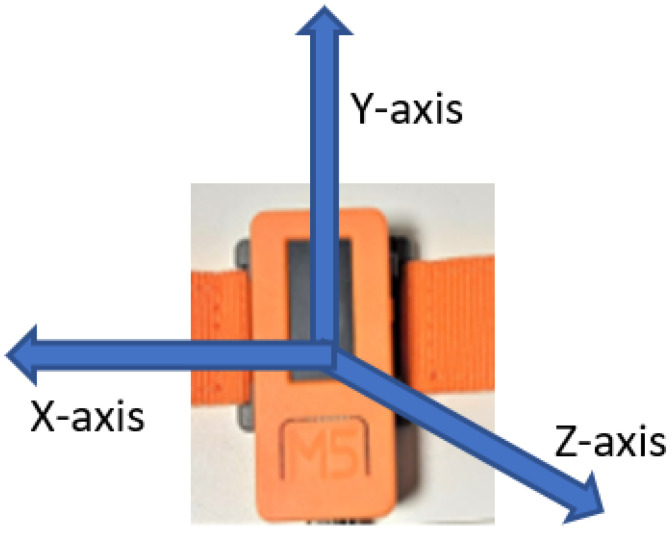
Axes presentation on the accelerometer device.

The wearable device M5-StickC [35] was equipped on the chest to acquire acceleration data from 5 people. The utilized sensor was a triple-axis MEMS accelerometer in MPU6886. A digital output X-, Y-, and Z-axis accelerometer with programmable full-scale range of ±2 g, ±4 g, ±8 g and ±16 g and integrated 16-bit analog-to-digital Converter was used. For practical purposes in real-time, the data frequency was about 10 Hz because higher frequencies consume more battery energy, requiring more frequent device charging. The X-axis, Y-axis, Z-axes of the accelerometer device are represented in Figure 2. 

### 3.2. Input Features

The accelerometer data were acquired and saved to the text file for training. The provided data from the accelerometer were Xacc, Yacc, and Zacc, which are the acceleration of the X-axis, Y-axis, and Z-axis, respectively. From these data, other features were calculated before being used in ML models. Totally, there were 9 features in operation, as described below: [Xacc, Yacc, Zacc, ΔXacc, ΔYacc, ΔZacc, Δacc_Norm, Δ Roll, Δ Pitch] as shown in Table 1, where:

$$\Delta \mathrm{acc}\_\mathrm{Norm}=\sqrt{\Delta Xacc^{2}+\Delta Yacc^{2}+\Delta Zacc^{2}}$$

### 3.3. Regular ML Model for Classification

**Figure 3:**
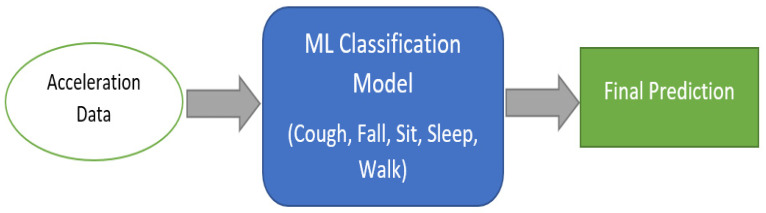
Regular ML classification model.

In the traditional method, the ML model trains all types of activities in one model. Five behavior categories were fed into the ML model for the training and validation process. Once the model was ready, it was capable of predicting the acceleration inputs, as illustrated in Figure 3. This technique’s accuracy can meet the considerable challenge when the sensor data contain significant noise and random jumping values as spikes. 

### 3.4. PTLE System for Activity Recognition

#### 3.4.1. System Overview

**Figure 4:**
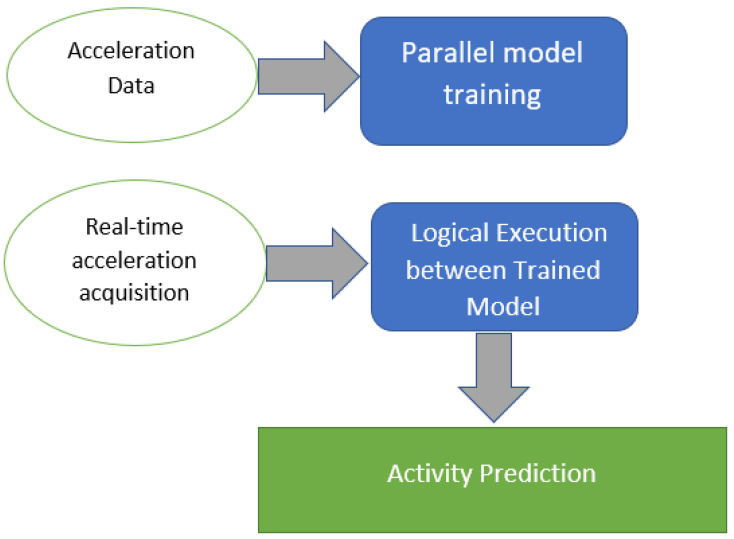
System overview diagram.

As shown in Figure 4, the total system contained 2 main stages. The first stage was about the training model by ML algorithms with accelerations. In the second stage, all models were well-trained and converted the real-time acceleration into the corresponding activity based on the logical execution. 

#### 3.4.2. Parallel Training Models

**Figure 5:**
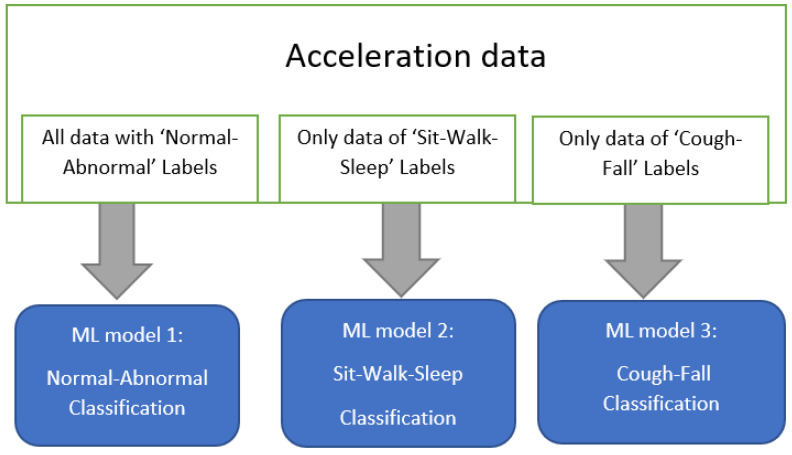
Parallel training models.

In this case, the PTLE system contained 3 ML models under training in parallel as shown in Figure 5. The first model included all data labeled as ‘normal’ and ‘abnormal’ activities. The second model only trained the ‘sit’, ‘walk’ and ‘sleep’ types. The third model trained with ‘cough’ and ‘fall’ data. 

With this kind of task division, each model will focus more on the specific categories instead of working on all activities simultaneously. The cough is essential to detect, so when the user coughs during walking, sitting, or sleeping, the data are still in the cough category. 

#### 3.4.3. Parallel Training Models

**Figure 6:**
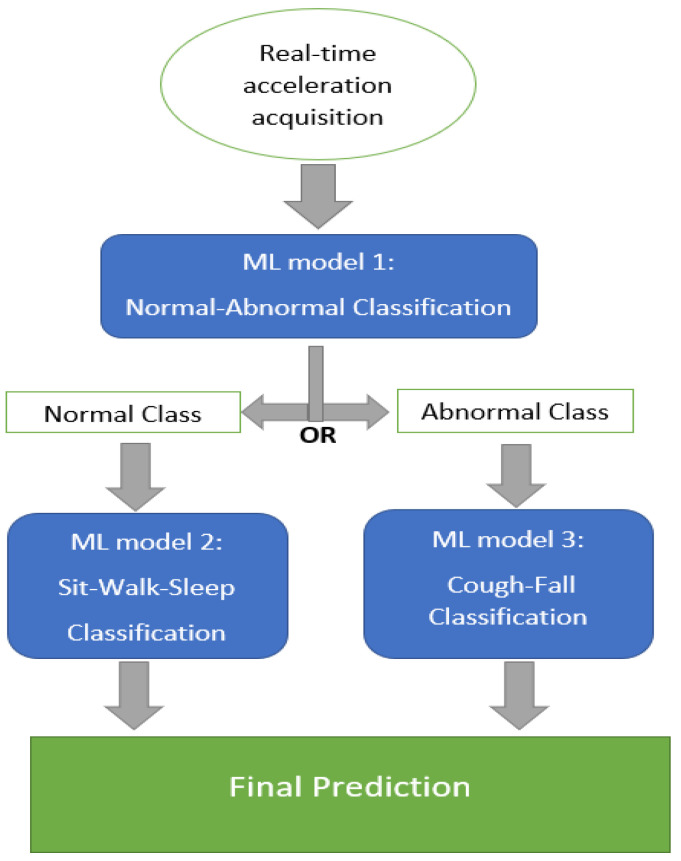
Logical execution chart of the PTLE system.

After the training process, three models were put to work following the propagation of logical execution. The mission of the first model was to separate abnormal symptoms from normal activities. Once the abnormal class was detected, the PTLE system passed the data into the third model, which distinguished between cough and fall. In another case, the data come to the second model, and sit, walk, or sleep was recognized therein, as described in Figure 6. 

### 3.5. Suitable Algorithm Selection

At this stage, the most fitting algorithm has to be selected. The function K-fold and cross-validation were utilized to evaluate the ML algorithms. Cross-validation is a resampling procedure used to evaluate machine learning models on a limited data sample.

The procedure contains a single parameter called K, which refers to the number of groups into which a given data sample will be split. This approach involves randomly dividing the set of observations into K groups, or folds, of approximately equal size. The first fold is treated as a validation set, and the method fits the remaining K − 1 folds. In this case, 10 K-folds were used in cross-validation—the score was in the range of 0 to 1. The ML model had a higher score and achieved better accuracy. 

As described in Section 2, seven popular algorithms of ML classification were compared with each other to determine the most suitable algorithm for this particular circumstance. 

Table 2 reports the mean score and standard deviation (Std) score of 10-fold validation for model 1 (normal–abnormal). As a result, the RF obtained the best score with less variation than the other algorithms, so this algorithm was chosen as the primary ML model for the activity classification. The RF hypermeters constitute 100 trees in the forest; 1 leaf minimum sample is needed to be at a leaf node, and 2 minimum sample splits are required to split an internal node. Nodes were expanded until all leaves contained less than the minimum sample splits.

**Figure 7:**
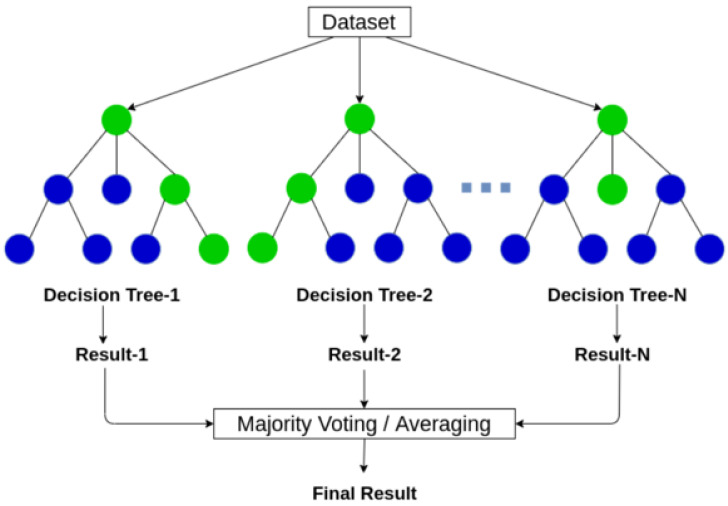
RF structure [37].

RF changes the algorithm in terms of how the sub-trees are learned so that the resulting predictions from all subtrees have less correlation using the bootstrap method, as demonstrated in Figure 7. In each bootstrap training set, about one-third of the instances enter each decision tree [36]. RF is an ensemble of decision trees that includes 3 main features for data processing:

- Every single tree is constructed of a different sample of rows. At each node, a different sample of features is selected for splitting to the following stages.
- Each of the trees provides its prediction.
- These predictions are averaged to supply the last decision value based in the tree outputs.

**Figure 8:**
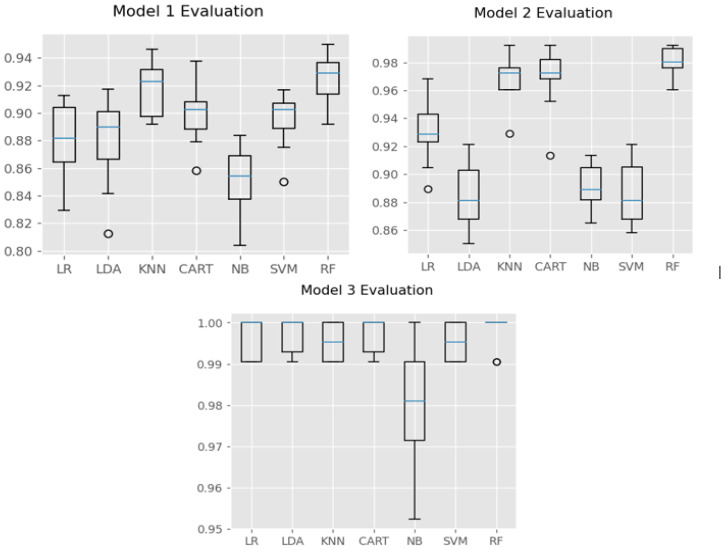
Chart of algorithm evaluation in the three models.

As shown in Figure 8, RF worked well with all 3 models in PTLE system. As reported above, model 1 surpassed the other algorithms with the highest accuracy after 10-fold validation. In models 2 and 3, RF still achieved the most impressive results with high mean and less Std, even though CART and KNN were also good candidates. Therefore, the RF was selected as the utilized algorithm for all 3 models to classify the activities. 

### 3.6. Performance Validation

To validate the proposed technique, the following ML factors were calculated: precision, recall, and F1-Score based on true positive (TP_A_), false positive (FP_A_), and false negative (FN_A_) of class A.

- TP_A_ is the number of predictions where the classifier correctly predicts class a.
- FP_A_ is the number of objects that do not belong to class a but are predicted as class a.
- FN_A_ is the number of objects from class A predicted to another class.
- Precision quantifies the number of positive class predictions that actually belong to the positive class. It is calculated as the sum of true positives across all classes divided by the sum of true positives and false positives across all classes.

(1) $$\mathrm{Precision}=\frac{TP_{A}}{TP_{A}+FP_{A}}$$

- Recall quantifies the number of positive class predictions made from all positive examples in the dataset. Unlike precision, that only comments on the correct positive predictions out of all positive predictions, recall provides an indication of missed positive predictions. In multiple classification, recall is determined as the sum of true positives across all types divided by the sum of true positives and false negatives across all categories.

(2) $$\mathrm{Recall}=\frac{TP_{a}}{TP_{A}+FN_{A}}$$

- F1-Score provides a single score that balances the concerns of precision and recall in one number. F-Score delivers a way to combine both precision and recall into a single measure that captures both properties. Once precision and recall have been calculated for the multiclass classification problem, the two scores can be combined into the calculation of the F-Measure. As with precision and recall, a poor F-Measure score is 0.0, and a best or perfect F-Measure score is 1.0

(3) $$F1-\mathrm{Score}=\frac{2*\mathrm{Precision}*\mathrm{Recall}}{\mathrm{Precision}+\mathrm{Recall}}$$

- Accuracy is the fraction between number of correct predictions and number of predictions.

(4) $$\mathrm{Accuracy}=\frac{\mathrm{Correct}\;\mathrm{predictions}}{\mathrm{Total}\;\mathrm{predictions}}$$

For training and testing process, the user wore the device on their chest, which sent the accelerations to the PC via Wi-Fi. Table 3 demonstrates the validation result of the PTLE method on the testing sample, which is about 30% of the total collected samples; the rest of the samples were used to train the model. The system showed a balance of prediction with a good F1-Score and overall accuracy of 99%. 

## 4. Experiments and Results

### 4.1. Real-time Test Validation

In the experiment, two different users equipped the M5-StickC on their chests; one was an old person, and another had just recovered from the flu. These users performed activities following the indication during a specific period. The fall events were acquired with the support of a volunteer group. 

The M5stickC is a development platform that includes flash memory of 4 MB and a Lipo-battery of 95 mA-3.7 V, which the Arduino Software (IDE) [38] can program. This board contains the MEMS 3-axis accelerometer MPU6886. The acceleration range is ±2 g in this application. 

ML models were designed in Python based on scikit-learn [39], a powerful and easy-to-use free, open-source Python library for ML development and evaluation. The models were operated by a PC processor: Intel(R) Core (TM) i7-10850H CPU @ 2.70 GHz.

This wearable device acquired the sensor data, which were sent to the ML model on PC via Wi-Fi based on MQTT protocol via Ubidots software [40] which performs functions such as IoT data analysis and visualization. The ML models received these data and performed the activity prediction. Each action was carried out for a period and the predictions were verified in real-time, with the ground truth being the current activity. 

Here, the regular ML mode with the RF algorithm was designed, where a single model was trained to classify all five activities. The comparison between the two methods was demonstrated to analyze the efficiency of each approach. There were numerous numbers of samples acquired for ML model validation, as reported in Table 4. The regular model had significant difficulty in making the prediction for sleep. The accelerometer frame collected data in terms of the X–Y–Z axis during activities such as lying on the bed and other activities that can cause confusion for the regular ML model. Meanwhile, the PTLE technique was still able to classify sleep well, with high precision and good recall. 

Regarding sit and walk, the regular model could achieve better prediction than sleep, with F1 scores being acceptable, but it was still inferior to the proposed system. The PTLE system showed that the prediction balance with precision and recall were always higher than 0.80.

Cough and fall are the two most crucial categories to detect. The regular model prediction was average at this point. Low precision in cough means that the model misunderstands other behaviors such as cough. In contrast, this ML model improperly predicted many fall cases as other behaviors brought uncertainty to the results. With more specific training, the PTLE system could accomplishes stable prediction with high efficiency. Although fall recall is still not uppermost, the fall prediction is already optimized significantly with absolute precision and a high F1 score. The PTLE system had a good performance in previous tests during the training process. Obviously, there was slight difference in the results between the two tests because the data were obtained from different users at different periods. 

### 4.2. Bar Chart Comparision of Correct Prediction Number 

**Figure 9:**
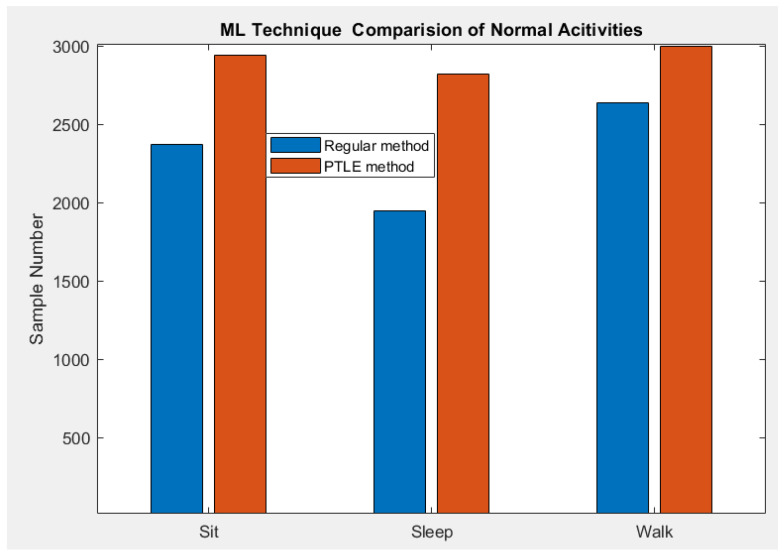
Correct prediction from the two methods in normal activities.

**Figure 10:**
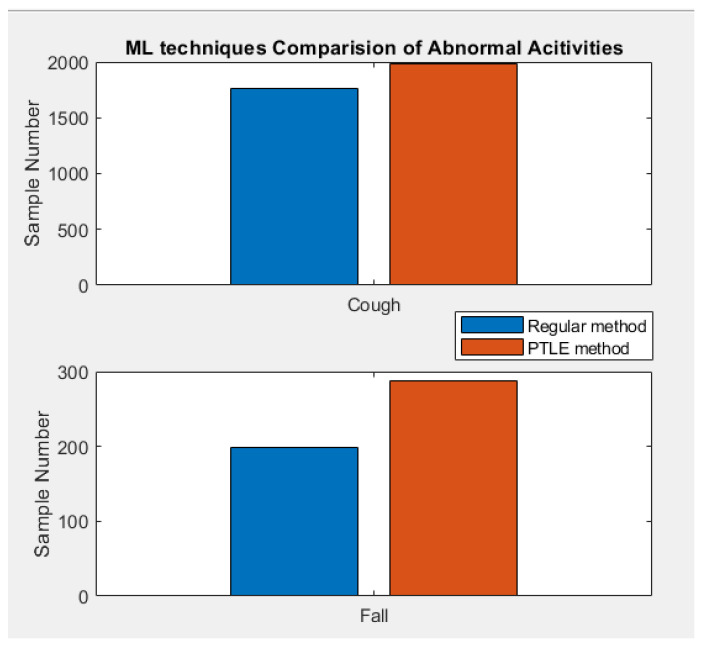
Correct prediction from the two methods in abnormal activities.

Figure 9 and Figure 10 illustrate the correct number of prediction samples from both methods in each activity. In normal activities, PTLE method provided more than 10% of precise decisions compared with the regular method, which indicates the superior operation based on the logical execution after the parallel training for the models.

Regarding abnormal activity detection, cough was recognized very well by the PTLE system. Its correct prediction was almost absolute, which is a huge advantage since this symptom is important to realize for weak people. The fall recognition was also reliable, with approximately 98% of proper outputs. Thus, the PTLE method is satisfactory for tracking abnormal behaviors of weak people in real-time. 

Table 5 reports the standard deviation (Std) and the prediction accuracy of the correct prediction from the two models. In normal activities, the Std of PTLE was less than the regular model and vice-versa in the abnormal case. Regarding the accuracy, the PTLE accomplished a superior number of correct predictions in both situations. 

### 4.3. Activity Visulization on IoT Dashboard 

**Figure 11:**
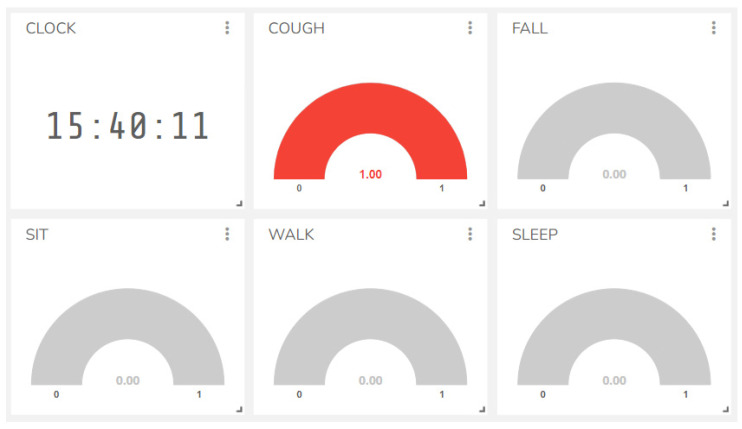
Activity prediction in real time on the IoT dashboard.

The activity prediction were sent to the Ubidots dashboard with two logic values: 0—No (no colour); 1—Yes (red colour) as shown in Figure 11. In this example, the user coughed, so the cough box assumed a red colour with a logic of 1 and the other action box had no colour. During sit, walk, or sleep, if the user coughed, the ML system would predict cough over the others. 

Number of coughs per day can be calculated and observed in the website history. Regarding the fall events, once they occur, they will remain as logic 1 with a red color as an alarm to the observers until the user presses the restart button on the wearable device or there is a manual intervention from the responsible website/PC control. During this fall event, the other activities are still under normal prediction of the ML system. Additionally, the observers can understand the health state and the emergency level of the user.

## 5. Conclusions

The paper described a new system classification based on ML models to optimize the prediction quality for the risk factors of health behavior for weak people. The PTLE system managed the trained models following logical operation, which reduced the complexity of each ML model and upgraded the output accuracy. In addition, the proposed idea enables a new approach of multiple-algorithm combination per ML structure in one training flow. Moreover, the project utilized a low-cost and small-size device conveniently equipped with to body for supervising vital activities, cough and fall, from other normal actions. An IoT protocol was implemented to support communication between the sensor and workstation with the MQTT and cloud that help the responsible people monitor the vulnerable person at a distance.

In the future, the designed system will be tested with more people to learn more about the pros and cons of the system. Obtained measurements from various people can support the project in developing the device in terms of prediction precision and signal communication, which are extremely useful not only in the case of medical care but also in HAR generally. 

## Figures and Tables

**Table 1:** ML inputs and output.

Inputs									Output
Xacc	Yacc	Zacc	ΔXacc	ΔYacc	ΔZacc	Δacc_Norm	Δ Roll	Δ Pitch	Activity

**Table 2:** Performance validation for ML algorithms.

Algorithm	Mean Score	Std Score
RF	0.931	0.009
KNN	0.918	0.015
CART	0.907	0.0126
SVM	0.899	0.0164
LR	0.889	0.014
LDA	0.888	0.0182
NB	0.857	0.0193

**Table 3:** PTLE validation.

Activity	Precision	Recall	F1-Score	Support Sample
Cough	0.96	0.99	0.98	1688
Fall	1.00	0.98	0.99	200
Sit	0.99	0.93	0.96	1500
Sleep	1.00	0.92	0.96	1500
Walk	0.95	0.96	0.96	1500
Total sample: 4000 Overall Accuracy: 0.99				

**Table 4:** Regular model and PTLE validation.

ML Method	Activity	Precision	Recall	F1-Score	Support Sample
Regular Model	Cough	0.61	0.88	0.72	2000
	Fall	0.78	0.66	0.71	300
	Sit	1.00	0.79	0.88	3000
	Sleep	0.59	0.65	0.62	3000
	Walk	0.64	0.88	0.74	3000
	Overall Accuracy: 0.79				
PTLE System	Cough	0.98	0.99	0.98	2000
	Fall	1.00	0.96	0.98	300
	Sit	1.00	0.98	0.99	3000
	Sleep	1.00	0.94	0.97	3000
	Walk	0.88	1.00	0.94	3000
	Overall Accuracy: 0.98				

**Table 5:** Correct prediction comparison metrics.

	Parameters	Regular Model	PTLE Model
Std (Samples)	Normal activity	781	846
	Abnormal activity	283.9	74.83
Prediction accuracy (%)	Normal activity	85.13	98.6
	Abnormal activity	77.33	97.34

## References

[1] World Health Organization Coronavirus Disease (COVID-19). https://www.who.int/emergencies/diseases/novel-coronavirus-2019.

[2] Tufts Medical Center. https://hhma.org/can-a-cough-damage-my-lungs/.

[3] Zakaria N.A., Kuwae Y., Tamura T., Minato K., Kanaya S. (2013). Quantitative analysis of fall risk using TUG test. Comput. Meth. Biomech. Biomed. Eng..

[4] Long H.M., Pietrosanto A. (2020). A Robust Orientation System for Inclinometer with Full-Redundancy in Heavy Industry. IEEE Sens. J..

[5] Long H.M., Iacono S.D., Paciello V., Pietrosanto A. (2021). Measurement Optimization for Orientation Tracking Based on No Motion No Integration Technique. IEEE Trans. Instrum. Meas..

[6] Long H.M., Carratu M., Ugwiri M.A., Paciello V., Pietrosanto A. A New Technique for Optimization of Linear Displacement Measurement Based on MEMS Accelerometer. Proceedings of the 2020 International Semiconductor Conference (CAS).

[7] Kang J., Shin J., Shin J., Lee D., Choi A. (2022). Robust Human Activity Recognition by Integrating Image and Accelerometer Sensor Data Using Deep Fusion Network. Sensors.

[8] Khan Y.A., Imaduddin S., Prabhat R., Wajid M. Classification of Human Motion Activities using Mobile Phone Sensors and Deep Learning Model. Proceedings of the 2022 8th International Conference on Advanced Computing and Communication Systems (ICACCS).

[9] Zebin T., Scully P., Ozanyan K.B. Evaluation of supervised classification algorithms for human activity recognition with inertial sensors. Proceedings of the 2017 IEEE Sensors.

[10] Tian Y., Chen W. MEMS-based human activity recognition using smartphone. Proceedings of the 2016 35th Chinese Control Conference (CCC).

[11] Logacjov A., Bach K., Kongsvold A., Bårdstu H.B., Mork P.J. (2021). HARTH: A Human Activity Recognition Dataset for Machine Learning. Sensors.

[12] Stewart T., Narayanan A., Hedayatrad L., Neville J., Mackay L., Duncan S. (2018). A Dual-Accelerometer System for Classifying Physical Activity in Children and Adults. Med. Sci. Sport Exerc..

[13] Cleland I., Kikhia B., Nugent C., Boytsov A., Hallberg J., Synnes K., McClean S., Finlay D. (2013). Optimal Placement of Accelerometers for the Detection of Everyday Activities. Sensors.

[14] Bao L., Intille S.S., Ferscha A., Mattern F. (2004). Activity Recognition from User-Annotated Acceleration Data. Pervasive Computing.

[15] Olguín D.O., Pentland A. Human activity recognition: Accuracy across common locations for wearable sensors. Proceedings of the IEEE 10th International Symposium on Wearable Computers.

[16] Nachiar C.C., Ambika N., Moulika R., Poovendran R. Design of Cost-Effective Wearable Sensors with Integrated Health Monitoring System. Proceedings of the 2020 Fourth International Conference on I-SMAC (IoT in Social, Mobile, Analytics and Cloud) (I-SMAC).

[17] Abid M., Khabou A., Ouakrim Y., Watel H., Chemcki S., Mitiche A., Benazza-Benyahia A., Mezghani N. (2021). Physical Activity Recognition Based on a Parallel Approach for an Ensemble of Machine Learning and Deep Learning Classifiers. Sensors.

[18] Hoang M.L., Pietrosanto A. (2022). New Artificial Intelligence Approach to Inclination Measurement Based on MEMS Accelerometer. IEEE Trans. Artif. Intell..

[19] Hoang M.L., Pietrosanto A. (2021). Yaw/Heading Optimization by Drift Elimination on MEMS Gyroscope. Sens. Actuators A Phys..

[20] Long H.M., Iacono S.D., Paciello V., Pietrosanto A. Pre-Processing Technique for Compass-Less Madgwick in Heading Estimation for Industry 4.0. Proceedings of the 2020 IEEE International Instrumentation and Measurement Technology Conference (I2MTC).

[21] Long H.M., Pietrosanto A. An Effective Method on Vibration Immunity for Inclinometer Based on MEMS Accelerometer. Proceedings of the 2020 International Semiconductor Conference (CAS).

[22] Hu R., Michel B., Russo D., Mora N., Matrella G., Ciampolini P., Cocchi F., Montanari E., Nunziata S., Brunschwiler T. (2021). An Unsupervised Behavioral Modeling and Alerting System Based on Passive Sensing for Elderly Care. Future Internet.

[23] Tapia E.M., Intille S.S., Haskell W., Larson K., Wright J., King A., Friedman R. Real-time recognition of physical activities and their intensities using wireless accelerometers and a heart monitor. Proceedings of the International Symposium on Wearable Computers.

[24] Parkka J., Ermes M., Korpipaa P., Mäntyjärvi J., Peltola J., Korhonen I. (2006). Activity Classification Using Realistic Data from Wearable Sensors. IEEE Trans. Inf. Technol. Biomed..

[25] Mekruksavanich S., Hnoohom N., Jitpattanakul A. Smartwatch-based sitting detection with human activity recognition for office workers syndrome. Proceedings of the 2018 International ECTI Northern Section Conference on Electrical, Electronics, Computer and Telecommunications Engineering (ECTI-NCON).

[26] Kwon M.-C., Choi S. (2018). Recognition of daily human activity using an artificial neural network and smartwatch. Wirel. Commun. Mob. Comput..

[27] Paul P., George T. An effective approach for human activity recognition on smartphone. Proceedings of the 2015 IEEE International Conference on Engineering and Technology (ICETECH).

[28] Hoang M.L., Pietrosanto A. (2022). Yaw/Heading optimization by Machine learning model based on MEMS magnetometer under harsh conditions. Measurement.

[29] Vedaei S.S., Fotovvat A., Mohebbian M.R., Rahman G.M.E., Wahid K.A., Babyn P., Marateb H.R., Mansourian M., Sami R. (2020). COVID-SAFE: An IoT-Based System for Automated Health Monitoring and Surveillance in Post-Pandemic Life. IEEE Access.

[30] Yin Y., Zeng Y., Chen X., Fan Y. (2016). The internet of things in healthcare: An overview. J. Ind. Inf. Integr..

[31] Priyamvadaa R. Temperature and Saturation Level Monitoring System Using MQTT for COVID-19. Proceedings of the 2020 International Conference on Recent Trends on Electronics, Information, Communication & Technology (RTEICT).

[32] Yuan H., Wang Z., Xia L. Design of Temperature and Humidity Detection System for a Material Warehouse Based on GM. Proceedings of the 2020 IEEE 4th Information Technology, Networking, Electronic and Automation Control Conference (ITNEC).

[33] Hoang M.L., Carratù M., Paciello V., Pietrosanto A. (2021). Body Temperature—Indoor Condition Monitor and Activity Recognition by MEMS Accelerometer Based on IoT-Alert System for People in Quarantine Due to COVID-19. Sensors.

[34] Espressif Systems (Shanghai) Co., Ltd (2020). ESP32 Series Datasheet, Version 3.4.

[35] Hackster.io M5Stack M5StickC ESP32-PICO Mini IoT Development Board Projects. https://m5stack.hackster.

[36] Breiman L. (2001). Random Forests. Mach. Learn..

[37] Sharma A. Decision Tree vs. Random Forest—Which Algorithm Should You Use? Analytics Vidhya, 12 May 2020. https://www.analyticsvidhya.com/blog/2020/05/decision-tree-vs-random-forest-algorithm/.

[38] Arduino Team “Software.” Arduino. https://www.arduino.cc/en/software.

[39] Pedregosa F., Varoquaux G., Gramfort A., Michel V., Thirion B., Grisel O., Blondel M., Prettenhofer P., Weiss R., Dubourg V. (2011). Scikit-learn: Machine Learning in Python. J. Mach. Learn. Res..

[40] Ubidots Data Drives Decisions. https://ubidots.com/.

